# Determination of Structural Requirements of *N*-Substituted Tetrahydro-β-Carboline Imidazolium Salt Derivatives Using in Silico Approaches for Designing MEK-1 Inhibitors

**DOI:** 10.3390/molecules22061020

**Published:** 2017-06-19

**Authors:** Jingwei Liang, Mingyang Wang, Xinyang Li, Xin He, Chong Cao, Fanhao Meng

**Affiliations:** School of Pharmacy, China Medical University, Liaoning 110122, China; liangjw89@163.com (J.L.); wmy940623@163.com (M.W.); 13125424162@163.com (X.L.); yhhexin@163.com (X.H.); caochong36@163.com (C.C.)

**Keywords:** 3D-QSAR, MEK-1, inhibitors, docking, molecular dynamics simulations

## Abstract

Novel *N*-substituted tetrahydro-β-carboline imidazolium salt derivatives proved to have potent antitumor activity in past research. The Topomer CoMFA and CoMSIA function in Sybyl-X 2.0 software was applied for the identification of important features of *N*-substituted tetrahydro-β-carboline-imidazolium salt derivative moieties. In the case of Topomer CoMFA, all the compounds were split into two fragments which were used to generate a 3D invariant representation, the statistical results of the Topomer CoMFA model: q^2^ value of 0.700; r^2^ value of 0.954; with 5 optimum components. The database alignment was utilized for building the CoMSIA model, and the CoMSIA model had q^2^ and r^2^ values of 0.615 and 0.897, with 4 optimum components. Target fishing of the PharmMapper platform was utilised for finding potential targets, the human mitogen-activated protein kinase 1 (MEK-1) was found to be the primary potential target for the three compounds with the fit scores of 6.288, 5.741, and 6.721. The molecular docking technique of MOE 2015 was carried out to identify the interactions of amino acids surrounding the ligand, and correlating QASR contour maps were used to identify structural requirements of *N*-substituted tetrahydro-β-carboline imidazolium salt moieties. Molecular dynamics and simulation studies proved that the target protein was stable for 0.8–5 ns. The pivotal moieties of *N*-substituted tetrahydro-β-carboline imidazolium salt derivatives and its potential targets were verified by the QASR study, PharmMapper, and the molecular docking study which would be helpful to design novel MEK-1 inhibitors for anticancer drugs.

## 1. Introduction

Cancer is the second leading cause of death globally, and was responsible for 8.8 million deaths in 2015. Globally, nearly 1 in 6 deaths is due to cancer [1]. The major goal of oncology scientists is to design an effective anticancer agent which is only sensitive in normal cancer cells, the ability to predict and alter, or block the hallmark of cancer cells is likely to improve the therapeutic index [2]. Therefore, the search for a targeted, effective drug with minimum toxicity is an urgent need [2,3].

**Figure 1:**
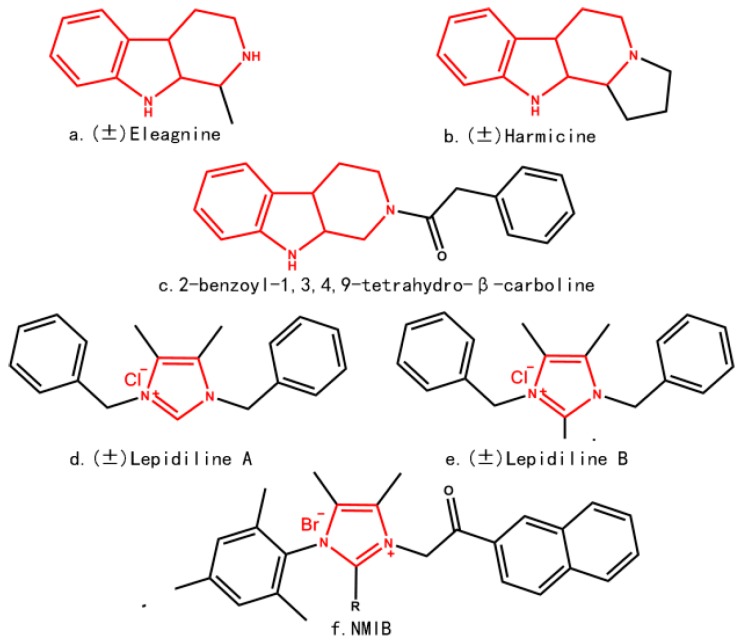
Representative alkaloids with *N*-substituted tetrahydro-β-carboline and imidazole moieties.

A series of novel *N*-substituted tetrahydro-β-carboline-imidazolium salt derivatives were designed and synthesized by using a molecular hybridization tool in past research [4], *N*-substituted tetrahydro-β-carboline-imidazolium salt derivatives were composed of *N*-substituted tetrahydro-β-carboline and imidazole moieties. Tetrahydro-β-carbolines consists of a large group of natural and synthetic alkaloids with the 9*H*-pyrido[3,4-*b*] indole being the common moiety, shown in Figure 1a–c. The tetrahydro-β-carbolines alkaloids have recently attracted attention due to its anti-HIV, anti-inflammatory, anti-leishmanial, anti-trypanosomal, and antitumor bioactivity [5,6,7,8]. Past research showed that these bioactivities are related to inhibition for some enzymes, such as kinesin spindle protein (KSP), monoamine oxidase (MAO), and mitogen activated protein kinase-activated protein kinase 2 (MAPKAPK2) [9,10,11]. Lepidiline A and B, NMIB, three novel imidazolium salts, shown in Figure 1d–f, have demonstrated the potent antitumor effects against human cancer cell lines [12,13]. In past research, the *N*-substituted tetrahydro-β-carboline-imidazolium salt derivatives with certain structures revealed potent cytotoxicity against HL-60, A-549, and MCF-7 cell lines [4].

Drug target identification is the important step in the drug discovery pipeline, PharmMapper is a freely accessed web-based tool, which is utilized for predicting the potential drug targets via a ‘reverse pharmacophore’ (also known as ‘target fishing’) mapping method [14]. Benefiting from the highly efficient and robust mapping method, PharmMapper with high-through-put ability can identify the potential target candidates from the database with a runtime of a few hours [14].

The RAS/RAF/MEK1/2/ERK1/2 signaling mitogen activated protein kinase (MAPK) cascade is an important signaling pathway in cancer involved in various cellular responses, including adaptation and survival [15,16]. MEK1 is the pivotal node in RAS/RAF/MEK1/2/ERK1/2 signaling cascades, which are responsible for the coordination and regulation of cancer cells growth and differentiation in response to extracellular stimulation [17]. Recent clinical data with MEK1/2 inhibitors have demonstrated the remarkable potential of targeting the signaling cascade for the treatment of certain cancers [18].

However, some MEK1 inhibitors are multikinase inhibitors that also inhibit the others kinase, so there is a lot of interest in finding more selective MEK1 inhibitors for specific targeted therapies [19]. Nevertheless, there has been no research of the detailed SAR and target in *N*-substituted tetrahydro-β-carboline imidazolium salt derivatives, although, ultimately tests in animals and humans are required, and there is a need to develop methods using in silico tests in order to decrease the time and cost of drug development [19]. So the ligand based QSAR techniques in Sybyl-X2.0 and the structure based docking study in MOE 2015 were applied to further research the relationship between the structural requirements and potential target. In this integrated study, the QSAR model and docking study in *N*-substituted tetrahydro-β-carboline-imidazolium salt derivates provided structural guidelines for designing selective MEK-1 inhibitors.

## 2. Results and Discussion

**Figure 2:**
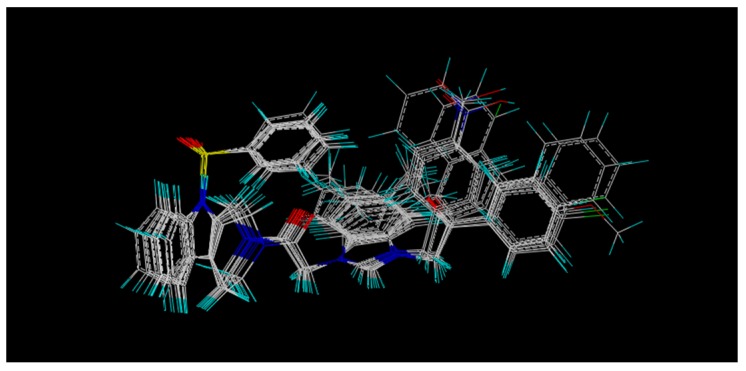
Superimposition of data set compounds for CoMSIA molecular field generation.

Under the fragment, statistical results of the Topomer CoMFA model: q^2^ value of 0.700; r^2^ value of 0.954; with 5 optimum components. The alignment results for the CoMSIA model is shown in Figure 2, the result of the database alignment was aligned over, and it also gave the best CoMSIA statistical parameters such as q^2^, r^2^, SEE, and F values as shown in Table 1. With the optimal number of components 4 in CoMSIA model, q^2^, r^2^, and SEE was found to be 0.615, 0.897, and 0.124, the statistical results proved that the QSAR model of Topomer CoMFA and CoMSIA have precise predictive predictability.

**Figure 3:**
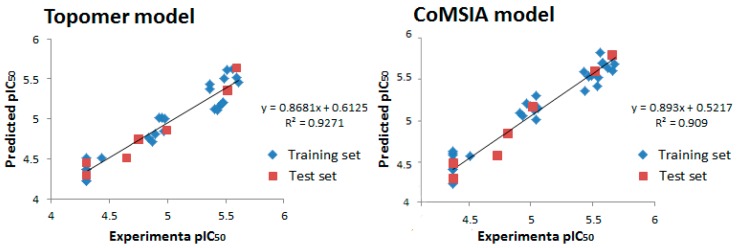
Experimental versus predicted activity of the training and test set based on the Topomer CoMFA model and CoMSIA model.

Experimental and predicted activities of both the training set and test set were shown in Figure 3, the Topomer CoMFA and CoMSIA model gave the correlation coefficient (r^2^) value of 0.9271 and 0.909, respectively, which demonstrated that the internal robustness and external high prediction of the QSAR models.

**Figure 4:**
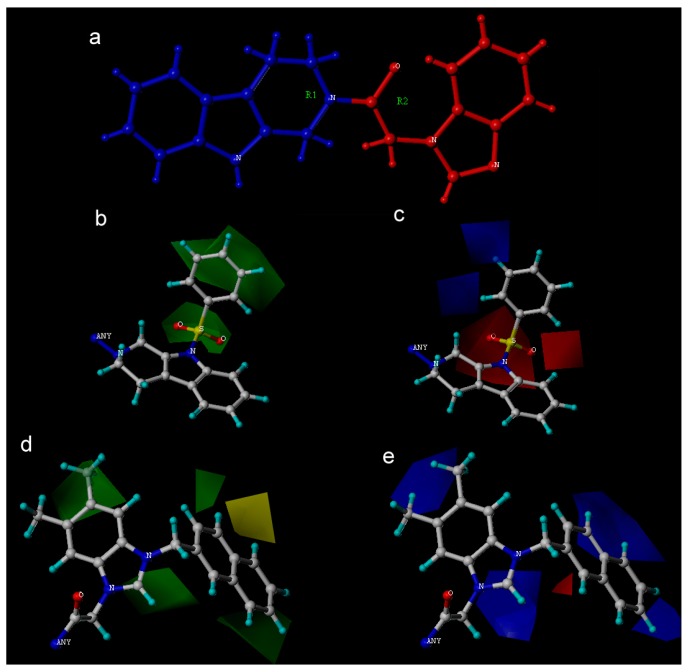
(**a**), Fragment 1 is blue, fragment 2 is red. Steric contour maps of Topomer CoMFA around R1 (**b**) and R2 (**c**), and electrostatic contour maps of Topomer CoMFA around R1 (**d**) and R2 (**e**) group. Green contours refer to sterically favourable regions and yellow contours refer to sterically unfavorable regions. Red contours refer to regions where electropositive groups are favorable and blue contours refer to regions where electropositive groups are unfavorable.

**Figure 5:**
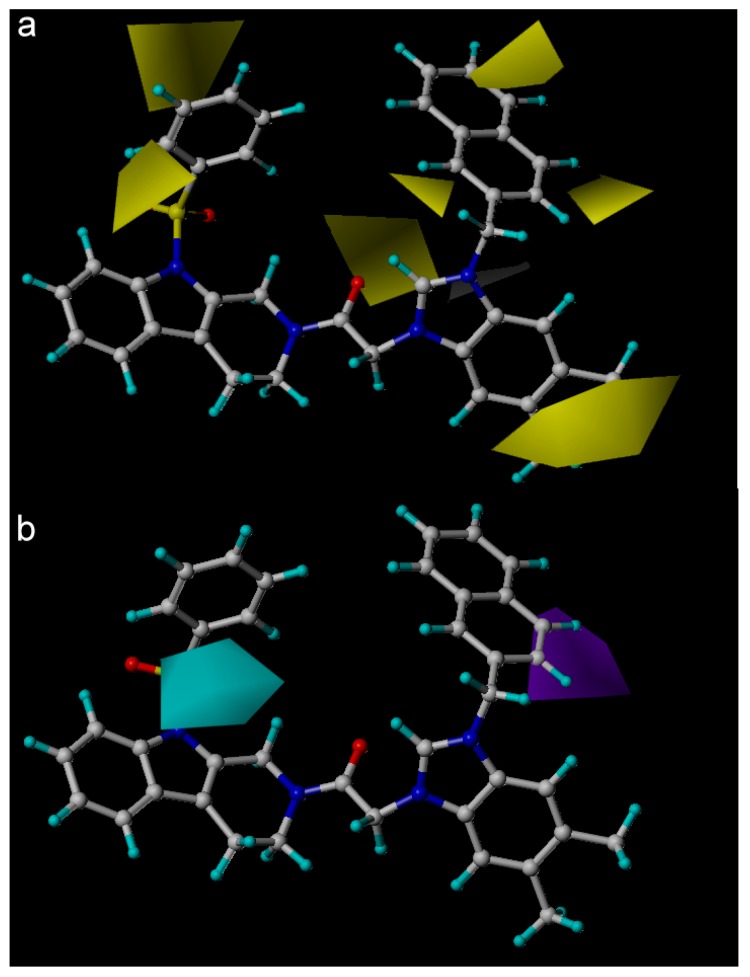
Hydrophobic (**a**) and H-bond acceptor (**b**) field contour maps for CoMSIA. Yellow contours represent regions where hydrophobic substituents are favorable, white contours represent regions where hydrophilic substituents are unfavorable, cyan contours indicate regions where hydrogen bond acceptor substituents increase activity, and purple contours indicate the unfavorable regions for hydrogen bond acceptor groups.

A 3D-QSAR contour map was utilized to exhibit the Topomer CoMFA and CoMSIA model properties lively in 3D-space, and to obtain the information in ligand-receptor conformation. The visualization of favorable and unfavorable regions of fields, namely, steric fields, electrostatic fields, hydrophobic fields, hydrogen bond donor atom fields, and hydrogen bond acceptor atom fields, contribute to realize the relationship between the compounds biological activity and structure. Transformation of the chemical group structure may increase or decrease the potency of the molecules. Steric and electrostatic contour maps of Topomer CoMFA QSAR model were shown in Figure 4b–e respectively. HBA and hydrophobic contour maps of CoMSIA QSAR model were shown in Figure 5a,b respectively. Compound **35** has the most complex chemical structure for the visual clarity of analyzing the QSAR, hence it was chosen as the reference structure for the generation of the Topomer CoMFA and CoMSIA contour map.

In the case of Topomer CoMFA analysis, the contour maps around fragment 1 and fragment 2 (Figure 4b–e) were generated from the Topomer CoMFA model. The steric contour map of fragment 1 was shown in Figure 4b, a green region near the *N*-substituents of the indole moiety, which proved that this R1 is sympathetic for sterically favorable functional groups, no yellow region was found in fragment 1. The electrostatic contour map of fragment 1 was shown in Figure 4c, combination of red region and blue region over the N-substituents of the indole moiety revealed that R1 is both sympathetic for electronegative and electropositive groups, and the R1 that was good for the anticancer activity can be regarded as a special part which N atoms-electronegative groups and electropositive groups connected together. The result can explain the fact that almost all of the compounds with the *N*-benzenesulfonylated substitution exhibited more potent inhibitory bioactivities than N-H imidazolium salts.

The steric contour map around fragment 2 was shown in Figure 4d, the green contour map neared the R2 and R3 of the imidazole ring suggested that the substitution of bulky groups will increase the antitumor activity, therefore the molecules have the scaffolds with 2-ethyl-imidazole (compounds **19**–**21**), and 5,6-dimethyl-benzimidazole (compounds **30**, **32,** and **35**) bulky groups showed significant activities. The green contour map also surrounded in the R4 of the imidazole ring suggested that the substitution of bulky groups such as naphthyl and substituted phenyl will increase the antitumor activity. This result can explain the fact that the compounds **24**, **25**, **26**, **30**, and **31** exhibited higher antitumor activity than the compound with small substituents or no substituents (compounds **20**–**22**, **24**, and **29**).

The sterically unfavorable yellow region was found over the substituents of R4 of imidazole ring, the carbonyl in the 3-position side chain of the imidazole ring turned the substituents direction, which is adverse to the activity, hence the compounds with higher activity (compounds **20**, **21**, **26**, and **35**) have no carbonyl in the 3-position of the imidazole ring.

As Figure 4e showed the electrostatic contour map around fragment 2, the blue region over the R2, R3, and R4 of the imidazole ring revealed that the placement of electropositive groups is in favor of anticancer activity, this can be proven by the fact that the compounds with the 2-ethyl-imidazole, 5,6-dimethyl-benzimidazole and naphthyl moieties, demonstrated higher bioactivity. The red region surrounding molecular scaffolds was not distinct, hence nitrobenzyl and bromophenacyl substituents in R4 didn’t demonstrate neither negative nor weak inhibitory activities.

The CoMSIA contour hydrophobic map was shown in Figure 5a, the yellow contour map in R1 of the indole moiety indicated the region where addition of the hydrophobic groups would increase the inhibitory bioactivity while white contour map in position-3 of the imidazole ring proved that the imidazolium salt is more potent than compounds with imidazole ring (compounds **2**–**5**), other yellow regions observed around R2, R3, and R4, these contours revealed that compounds with the combination of ethyl or 3,5-dimethyl-phenyl in benzimidazole ring and hydrophobic substitutes in R4 of imidazole ring exhibited activities better than compounds with no substitutes in these position. Compounds with such groups for example, compounds **21**, **31**, and **32**, with the substitutes in R2 or R3 of the imidazole have more potent activity than compounds **16**, **24**, and **25**, which didn’t have any substitutes.

The contour map of the CoMSIA hydrogen bond acceptor (HBA) is shown in Figure 5b, the purple region in the R4 of the imidazole ring represented the HBA atom substitution is adverse to the activity, while the cyan region in the R1 of the indole moiety indicated that the addition of the HBD atom is conducive to the activity. The CoMSIA HBA contour map can be validated by the fact that compounds with a carbonyl side chain exhibited higher bioactivity while sulfonyl substituents exhibited bioactivity well.

**Figure 6:**
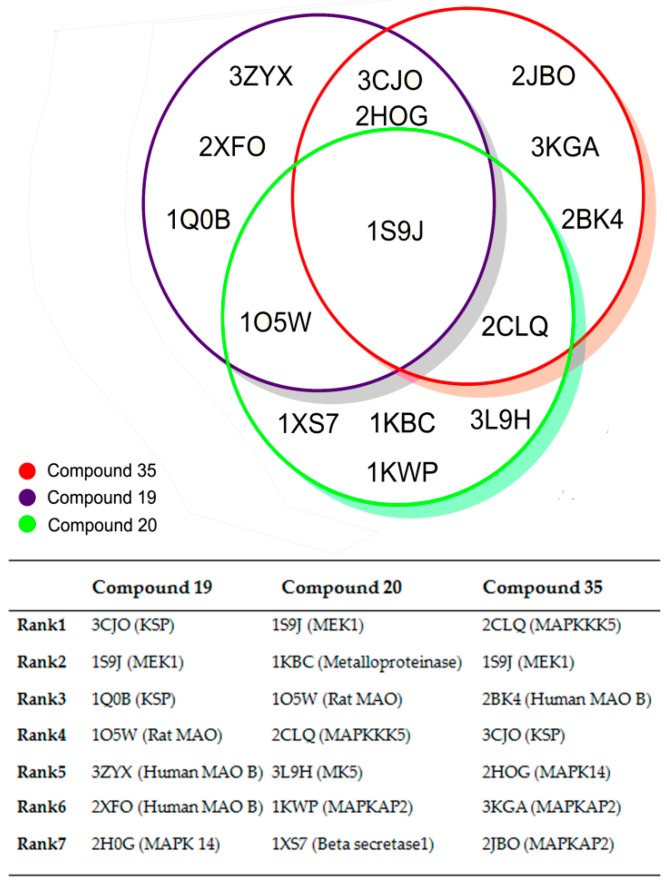
The result of the pharmMapper, 1s9j was the common target.

**Figure 7:**
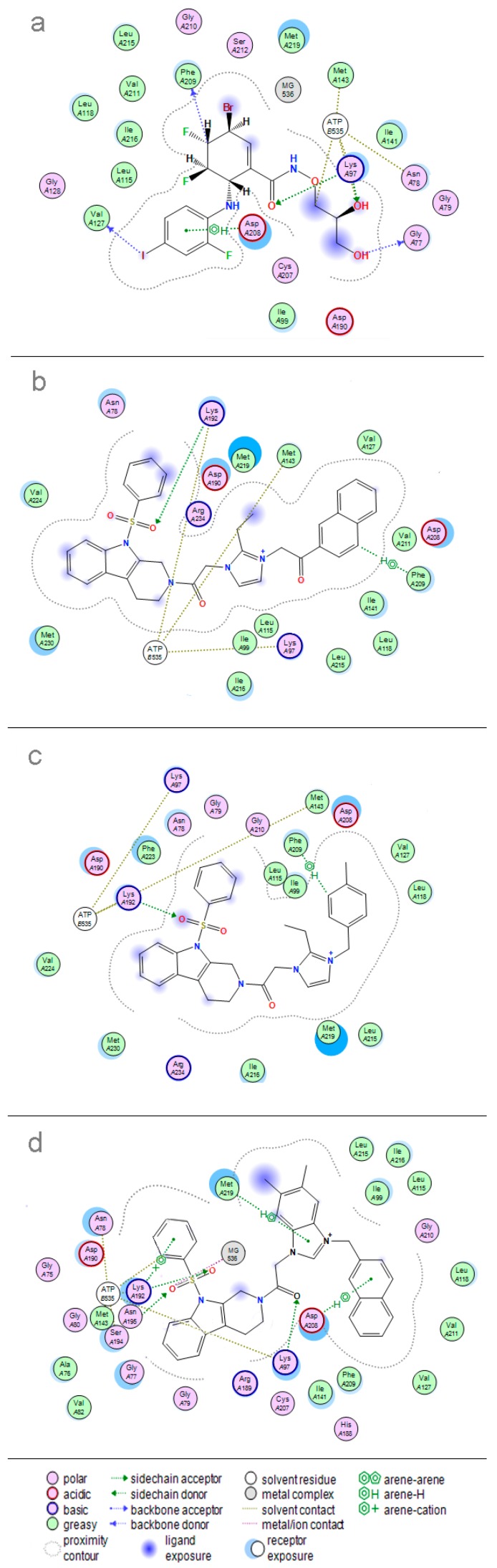
Molecular docking interactions of refametinib (**a**) and compounds **19** (**b**); **20** (**c**); and **35** (**d**).

In order to explore the mechanism of antitumor activity of *N*-substituted tetrahydro-β-carboline-imidazolium salt derivatives, we used the PharmMapper platform to predict their potential targets (in Figure 6). 1s9j was the common target, so MEK-1 (1s9j) was found to be the most potential protein target for compounds **19**, **20**, and **35** with the fit scores of 6.288, 5.791, and 6.721 in the PharmMapper platform. The past research showed that in the presence of inhibitors the MEK1 enzyme adopts the inactive conformation of helix C and the activation loop [20]. As the docking result of refametinib shown in Figure 7a, the noncompetitive MEK 1/2 inhibitor refametinib [21,22] binds in a pocket separate from, but adjacent to the Mg-ATP molecule site. ATP formed a solvent contact with Met143, Asn78, Lys97, and Lys192 instead of a normal binding mode with tyrosine and threonine residues when the ternary complex Mg-ATP-inhibitor exists in the binding site of the MEK-1 pocket. Therefore, the highly conserved glutamate residue Glu114 was unable to form a critical ion pair with the conserved catalytic Lys97 hence break through the rotation of helix C and the activation loop, and eventually occludes phosphorylation of ERK [20].

**Figure 8:**
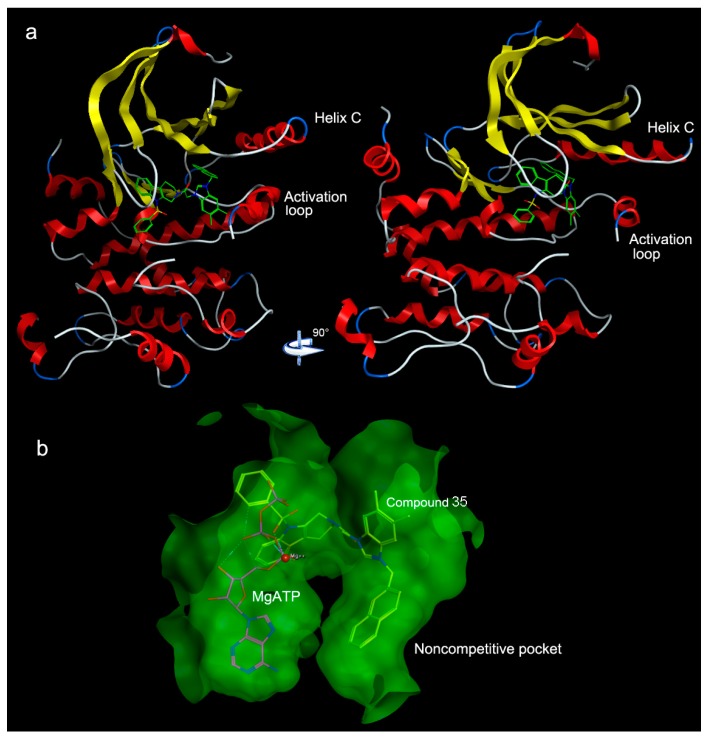
(**a**) Two views of the MEK1 protein kinase structure with the compound **36**; (**b**) 3D Views of the ternary complex of mitogen-activated protein kinase 1 (MEK-1) bound to compound **36** and MgATP in a noncompetitive pocket.

In the Figure 7, the structure of refametinib and compounds **19**, **20**, and **35** serve several similar significant interactions with amino acid residues around the binding pocket, refametinib formed many Van Der Waals interactions within the noncompetitive inhibitor pocket through amino acid residues such as Leu115, Asp208, Gly210, Phe209, Ser212, Ile141, Asp208, Lys97, and Asp190. We can assume that the binding mode of the refametinib and three compounds cause similar conformational changes in a noncompetitive inhibitor pocket near the helix C and activation loop by surrounding amino acids (Figure 8a).

The ligand-receptor interaction showed that the benzenesulfonylated substitution in compounds **19**, **20**, and **35** form an aromatic interaction with Lys192 (Figure 6b–d), in addition, the benzimidazole ring in compound **35** formed an aromatic interaction with Met219, naphthyl in the R4 substitution also showed aromatic interaction with Asp208 or Phe209. The docking result conforms to the Topomer CoMFA and CoMSIA contour maps, the Figure 4b and Figure 5a predicted that the bulky functional group will demonstrate the fine antitumor activity, the R1 substitution with benzenesulfonyl was well accommodated to the binding pocket. The basic amino acids Lys97 and Lys192 formed a hydrogen bond interaction to sulfonyl in Figure 5b, this result corresponded to the CoMSIA HBA contour map, where R1 was found to be favorable for the HBA group.

Negatively charged acidic amino acids Asp208 and Phe209 has an aromatic interaction with naphthyl in three compounds, which conform to the result of the Topomer CoMFA electrostatic contour map as shown in Figure 4e, where R4 substituents were found to be favorable for electropositive groups, moreover the docking study of three compounds showed that the bulky group, naphthyl, is suitable to the bottom of the binding pocket (compound **35** was selected as the representative in Figure 8b), it conforms to the Topomer CoMFA steric contour map. The dock study of three compounds demonstrated that R4 substitution was surrounded by greasy amino acids such as Ile141, Phe209, Val211, Val127, and Leu118 and it was mainly equal to the CoMSIA hydrophobic contour map. The hydrophobic group naphthyl or the substituted naphthyl in the R4 substitution have the possibility to increase the affinity to the surrounded amino acids. Thus, we can assess that the phenyl in R1 substitution and was not favorable for hydrophobic groups as phenyl was surrounded by polar amino acids, the hydrophilic substitution in phenyl may increase the affinity to the surrounding amino acids in pocket. The dock study showed that the benzimidazole ring formed hydrophobic interactions with greasy amino acids Leu215, Ile99, and Met219, which correspond to the Topomer CoMFA steric contour map where R2 and R3 of the benzimidazole ring were favorable to the steric groups such as methyl. 

**Figure 9:**
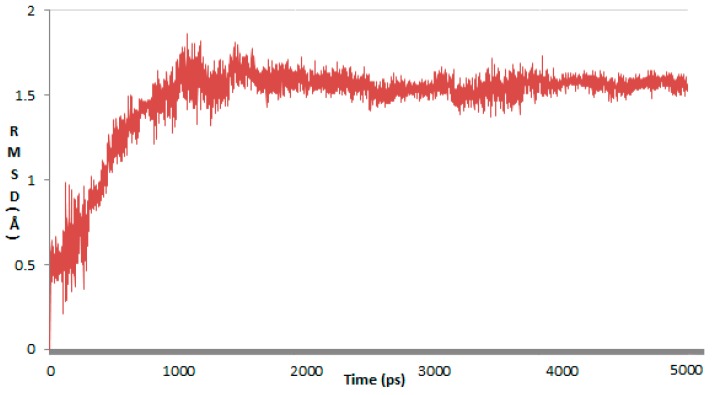
Root mean square deviation (RMSD) confirmations of protein-ligand complexes between 1 and 5 ns.

The root mean square deviations (RMSD) of the protein backbone with simulation time for the docked protein from the initial structure is shown in Figure 9, RMSD reached a value of 1.521 Å from 0.431 Å during 0–0.8 ns, and then retained between 1.502–2.195 Å throughout the simulation, and up to 5 ns for the docked protein. The averaged RMSD of the docked protein was found to be 1.522 Å, the MD/MS simulation indicated the docked protein stayed stability from 0.8 ns to 5 ns.

**Figure 10:**
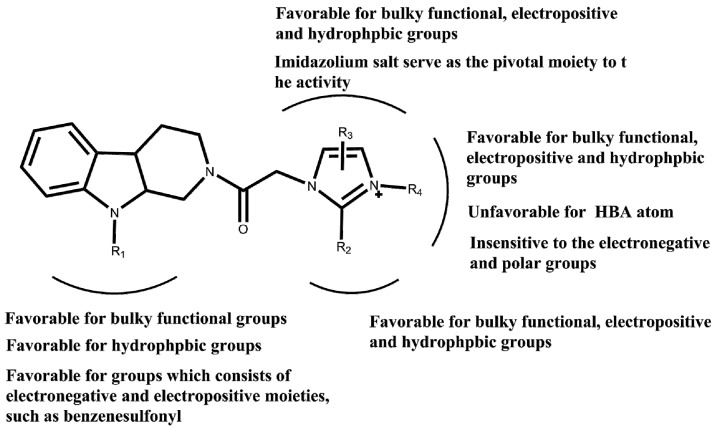
Important structural requirements of benzoxazepine moieties by means of the ligand based 3D-QSAR and structure based molecular docking study.

Finally, we combined the ligand based 3D-QSAR analysis with the structure based molecular docking study, to identify the necessary moiety of the *N*-substituted tetrahydro-β-carboline imidazolium salt derivatives (in Figure 10).

## 3. Materials and Methods

These 35 synthetic molecules were *N*-substituted tetrahydro-β-carboline-imidazolium salt derivatives that were selected from literature [4], all the synthetic molecules beared the tetrahydro-β-carboline moiety as shown in Table 2, the IC_50_ values against myeloid leukaemia (HL-60) cells, which was the most sensitive to the effect of the compounds, were converted into the corresponding pIC_50_, these pIC_50_ values were regarded as the dependent variable, while Topomer CoMFA and CoMSIA analyses were regarded as the independent variable. SKETCH function of Sybyl-X2.0 was utilized for drawing the structure and charges were calculated by the Gasteiger-Huckel method, and tripos force field was utilized for energy minimization of these molecules. These 35 synthetic molecules were divided into the training set and test set in the ratio of 80:20, the training set was used to build the 3D-QSAR model, and the test set was used to test the predictions of the model [23,24]. In the case of building the Topomer CoMFA QSAR model, a carbon sp^3^ probe was applied for calculating steric and electrostatic parameters. The topomer technique was applied to split the molecules into two fragments (the method of cutting is shown in Figure 4a) [25]. The database alignment was used to build the CoMSIA QSAR model [25], tetrahydro-β-carbolines was identified as the common core moiety.

Topomer CoMFA included steric and electrostatic fields for fragments, and CoMSIA included steric, electrostatic, hydrophobic, hydrogen bond donor (HBD) atom, and hydrogen bond acceptor (HBA) atom fields. PLS (partial least squares) techniques associated these field descriptors with the activity value [25]. Many statistics, such as values Leave One Out(LOO), optimal number of components, standard Error of Estimation (SEE), cross validated coefficients (q^2^), and conventional coefficient(r^2^), were important in the evaluation of the 3D-QSAR model, and could be worked out in the PLS method. The QSAR model is said to be good when the q^2^ value is more than 0.5 and the r^2^ value is more than 0.6, as q^2^ and r^2^ values reflect the model soundness, the best QSAR model depends on the highest q^2^ and r^2^ value, the lowest SEE, and optimal number of components [25]. In the case of Topomer CoMFA analysis, the PLS leave-one-out (LOO) method with CoMFA standard options for variable scaling was applied to investigate the Topomer CoMFA model [26].

In the CoMSIA analysis, the values of optimal number of components, SEE, q^2^ worked out by LOO validation, turning on USE SAMPLS, and components set to **5**. In the process of calculating r^2^, USE SAMPLS was turned off and column filtering set to 2.0 kcal·mo^−1^ to speed up the calculation without sacrificing information content [24,27], components set to **4**, which was the optimal number of components calculated by performing SAMPLS run. SEE and r^2^ were utilized to assess the non-cross validated models.

PharmMapper serves as a valuable tool for identifying potiential targets for a novel synthetic compound, a newly isolated natural product, a compound with known biological activity, or an existing drug whose mechanism of action is unknown [28].

Of all the *N*-substituted tetrahydro-β-carboline imidazolium salt derivatives in this research, compound **35** had exhibited bioactivity well, and was used to the potential prediction. The Mol2 format of compound **35** was submitted to the Pharmmapper server, the parameters of Generate Confomers and Maximum Generated Conformations was set as ON and 300, respectively, other parameters used the default values. 

The molecular docking study was carried out to find the binding mode and interactions between the amino acids in the protein's targeted pocket and the selective ligands by the software MOE2015. The refametinib and potential anticancer agents were carried out for the docking study. The crystal structures of the potential target was downloaded from the Protein Data Bank for the two ligands. Based on the London dG score, refametinib and compounds **19**, **20,** and **35** were docked into the noncompetitive inhibitor pocket of the MEK-1 protein by taking score values as the scoring function [29,30].

Preliminary MD simulations for the modeled protein were performed using the program NAMD (NAnoscale Molecular Dynamics program, v 2.9), and all files were generated using visual molecular dynamics (VMD). NAMD is a freely available software designed for high-performance simulation of large biomolecular systems [31]. During the MD simulation, the minimization and equilibration of docked protein in a 15 Å^3^ size water box, Amber12 EHT force field file was applied for energy minimization and equilibration with Gasteiger-Huckel charges using Boltzmann initial velocity [32]. The hydrogen atom coordinates of the docked protein were generated using the VMD Tk-Console salvation command. The physiological temperature and pressure was maintained at 310 K and 101.325 kPa, respectively. The cutoff distance for computing nonbonded interactions was truncated at 9 Å and long range electrostatic interactions were calculated using the particle-mesh Ewald (PME) method with an Ewald tolerance of 1e^−09^ [33]. The SHAKE algorithm applied to all bonds involving H-bonds [34]. Finally, we used 5 ns MD simulations, with a 2 fs timestep, performed to examine the root mean square deviations (RMSD) and hence investigated the stability of the protein-ligand complex.

## 4. Conclusions

MEK inhibitors bind to the inhibitory/allosteric segment adjacent to the ATP binding site, interfering with the enzyme kinase function in a highly specific and noncompetitive fashion. The results of the structure based docking study revealed that the non-competitive inhibitor refametinib and three compounds exhibit the similar binding mode which accommodate well in the non-competitive pocket. The 3D-QSAR study was utilized for the ligand based research, as it generates the reliable and predictive Topomer CoMFA and CoMSIA model, in Topomer CoMFA, the compound was split into two fragments, the value of q^2^ and r^2^ for the model is 0.700 and 0.954, the database alignment was found to be the best alignment method as it gave good statistical results with the q^2^ value of 0.615 and r^2^ value of 0.897. The 3D-QSAR study correlated with molecular docking study to reveal that R1 substitution is favorable for the functional groups which consisted of hydrogen bond acceptor atom, sterical groups, and hydrophilic terminal, R2 and R4 substitution is favorable for functional groups, which consisted of hydrophobic groups, electropositive groups, and sterical groups, on the other hand, electropositive groups and sterical groups existed in the imidazole ring and benzimidazole ring will have appropriate orientation in the noncompetive pocket, the HBA groups in the side chain of R3 is adverse to the activity. MD simulation was carried out to examine the stability of protein 1s9j, which revealed that the protein was forming a stable complex with ligands in 0.8–5 ns. Thus, the results of the ligand based research 3D-QSAR study, the structure based research molecular docking study, and the md simulation will be a guideline to design and synthesize the novel non-competitive MEK-1 inhibitors with the moiety of *N*-substituted tetrahydro-β-carboline and imidazole.

## Figures and Tables

**Table 1:** The PLS statistical parameters for the Topomer CoMFA and CoMSIA.

PLS Statistical Parameters	Topomer	CoMSIA
q^2 a^	0.700	0.615
r^2 b^	0.954	0.897
ONC ^c^	5	4
SEE ^d^	0.058	0.124
F ^e^	196.238	-
r_pred_^2 f^	0.914	0.852
Fraction of Field contributions ^g^		
Steric	-	0.245
Electrostatic	-	0.317
Hydrophobic	-	0.078
H-bond acceptor	-	0.124
H-bond donor	-	-

^a^ Cross-validated correlation coefficient; ^b^ Non-cross-validated correlation coefficient; ^c^ Optimum number of components; ^d^ Standard error of estimate; ^e^ F-test value; ^f^ The predictive r^2^ value; ^g^ Field: steric, electrostatic, hydrophobic, hydrogen-bond acceptor and hydrogen-bond donor.

**Table 2:**
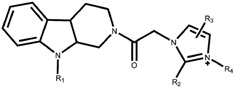
Structure of *N*-substituted tetrahydro-β-carboline imidazolium salt derivatives with the actual activity.

Compound	R_1_	R_2_	R_3_	R_4_	IC_50_ (μM)
**1**	H	H	Phenyl	-	>40
**2**	Benzenesulfonyl	H	-	-	>40
**3** *	Benzenesulfonyl	Ethyl	-	-	17.81
**4** *	Benzenesulfonyl	H	Phenyl	-	36.37
**5**	Benzenesulfonyl	H	3,5-dimethyl-phenyl		>40
**6**	H	H	-	benzoyl	>40
**7** *	H	H	-	2-naphthoyl	>40
**8**	H	H	Phenyl	benzoyl	>40
**9** *	H	H	Phenyl	4-methoxy benzoyl	>40
**10**	H	H	Phenyl	4-bromo benzoyl	21.81
**11**	H	H	Phenyl	2-naphthoyl	11.09
**12** *	H	H	Phenyl	4-bromo benzoyl	10.68
**13**	H	H	Phenyl	4-nitro benzoyl	>40
**14**	H	H	Phenyl	Naphthyl-2-methyl	3.54
**15**	Benzenesulfonyl	H	-	benzoyl	>40
**16**	Benzenesulfonyl	Ethyl	-	2-naphthoyl	3.32
**17**	Benzenesulfonyl	Ethyl	-	benzoyl	>40
**18**	Benzenesulfonyl	Ethyl	-	4-bromo benzoyl	11.87
**19**	Benzenesulfonyl	Ethyl	-	2-naphthoyl	2.47
**20**	Benzenesulfonyl	Ethyl	-	4-benzyl methyl	2.56
**21** *	Benzenesulfonyl	Ethyl	-	Naphthyl-2-methyl	2.77
**22**	Benzenesulfonyl	-	Phenyl	benzoyl	14.39
**23**	Benzenesulfonyl	-	Phenyl	4-bromo benzoyl	10.61
**24**	Benzenesulfonyl	-	Phenyl	4-methoxy benzoyl	3.97
**25**	Benzenesulfonyl	-	Phenyl	2-naphthoyl	3.24
**26** *	Benzenesulfonyl	-	Phenyl	4-benzyl methyl	3.04
**27**	Benzenesulfonyl	-	Phenyl	4-nitrobenzol methyl	13.58
**28**	Benzenesulfonyl	-	Phenyl	Naphthyl-2-methyl	4.34
**29**	Benzenesulfonyl	-	Phenyl	benzoyl	10.18
**30**	Benzenesulfonyl	-	3,5-dimethyl-phenyl	4-bromo benzoyl	3.75
**31**	Benzenesulfonyl	-	3,5-dimethyl-phenyl	4-methoxy benzoyl	3.39
**32**	Benzenesulfonyl	-	3,5-dimethyl-phenyl	2-naphthoyl	3.08
**33**	Benzenesulfonyl	-	3,5-dimethyl-phenyl	4-benzyl methyl	4.30
**34**	Benzenesulfonyl	-	3,5-dimethyl-phenyl	4-nitro-4-benzyl	12.81
**35**	Benzenesulfonyl	-	3,5-dimethyl-phenyl	Naphthyl-2-methyl	2.61

* Test set compound.

## References

[1] WHO Website. http://www.who.int.

[2] Yan Y., Caruso F. (2013). Particle carriers for combating multidrug resistant cancer. ACS Nano.

[3] Cholewinski G., Dzierzbicka K., Koodziejczyk M.M. (2011). Natural and syntheticacridines/acridones as antitumor agents: Their biological activities and methods of synthesis. Pharmacol. Rep..

[4] Zhou B., Liu Z.F., Deng G.G., Chen W., Li M., Yang L.J. (2016). Synthesis and antitumor activity of novel *n*-substituted tetrahydro-β-carboline imidazolium salt derivatives. Org. Biomol. Chem..

[5] Rook Y., Schmidtke K.U., Gaube F., Schepmann D., Wünsch B., Heilmann J. (2010). Bivalent β-carbolines as potential multitarget anti-alzheimer agents. J. Med. Chem..

[6] Lood C.S., Koskinen A.M.P. (2015). ChemInform Abstract: Harmicine, a Tetracyclic Tetrahydro-β-carboline: From the First Synthetic Precedent to Isolation from Natural Sources to Target-Oriented Synthesis (Review). Chem. Heterocycl. Compd..

[7] Vlahakis J.Z., Lazar C., Crandall I.E., Szarek W.A. (2010). Anti-plasmodium activity of imidazolium and triazolium salts. Bioorg. Med. Chem..

[8] Fortuna C., Barresi V., Berellini G., Musumarra G. (2008). Design and synthesis of trans, 2-(furan-2-yl)vinyl heteroaromatic iodides with antitumour activity. Bioorg. Med. Chem..

[9] Liu F., Yu L.Q., Jiang C., Yang L., Wu W.T., You Q.D. (2010). Discovery of tetrahydro-β-carbolines as inhibitors of the mitotic kinesin ksp. Bioorg. Med. Chem..

[10] Radulescu M.C., Bucur M.P., Bucur B., Radu G.L. (2015). Biosensor based on inhibition of monoamine oxidases A and B for detection of β-carbolines. Talanta.

[11] Trujillo J.I., Meyers M.J., Anderson D.R., Hegde S., Mahoney M.W., Vernier W.F. (2007). Novel tetrahydro-β-carboline-1-carboxylic acids as inhibitors of mitogen activated protein kinase-activated protein kinase 2 (mk-2). Bioorg. Med. Chem. Lett..

[12] Cui B., Zheng B.L., He K., Zheng Q.Y. (2003). Imidazole alkaloids from lepidium meyenii. J. Nat. Prod..

[13] Liu X., Ouyang S., Yu B., Liu Y., Huang K., Gong J., Zheng S., Li Z., Li H., Jiang H. (2010). PharmMapper Server: A web server for potential drug target identification via pharmacophore mapping approach. Nucleic Acids Res..

[14] Dhanasekaran N., Premkumar R.E. (1998). Signaling by dual specificity kinases. Oncogene.

[15] Kolch W. (2002). Ras/raf signaling and emerging pharmacotherapeutic targets. Expert Opin. Pharmacother..

[16] Zheng C.F., Guan K. (1993). Cloning and characterization of two distinct human extracellular signal-regulated kinase activator kinases, MEK1 and MEK2. J. Biol. Chem..

[17] Sale M.J., Cook S.J. (2014). Intrinsic and acquired resistance to MEK1/2 inhibitors in cancer. Biochem. Soc. Trans..

[18] Facciorusso A., Licinio R., Carr B.I., Di L.A., Barone M. (2015). Mek 1/2 inhibitors in the treatment of hepatocellular carcinoma. Expert Rev. Gastroenterol. Hepatol..

[19] Song W.J., Yang X.D., Zeng X.H., Xu X.L., Zhang G.L., Zhang H.B. (2012). Synthesis and cytotoxic activities of novel hybrid compounds of imidazole scaffold-based 2-substituted benzofurans. Rsc. Adv..

[20] Pauli I., Timmers L.F., Caceres R.A., Soares M.B., de Azevedo W.F. (2008). In silico and in vitro: Identifying new drugs. Curr. Drug Targets.

[21] Ohren J.F., Chen H., Pavlovsky A., Whitehead C., Zhang E., Kuffa P. (2004). Structures of human map kinase kinase 1 (mek1) and mek2 describe novel noncompetitive kinase inhibition. Nat. Struct. Mol. Biol..

[22] Lammers A., Weekes C.D. (2015). Refametinib: Dual MEK 1/2 inhibitor oncolytic. Drug Future.

[23] Lim H.Y., Heo J., Choi H.J., Lin C.Y., Yoon J.H., Hsu C. (2014). A phase II study of the efficacy and safety of the combination therapy of the mek inhibitor refametinib (bay 86–9766) plus sorafenib for Asian patients with unresectable hepatocellular carcinoma. Clin. Cancer Res..

[24] Klebe G., Abraham U., Mietzner T. (1994). Molecular similarity indices in a comparative analysis (comsia) of drug molecules to correlate and predict their biological activity. J. Med. Chem..

[25] Cichero E., Bruno O., Fossa P. (2012). Docking-based CoMFA and CoMSIA analyses of tetrahydro-β-carboline derivatives as type-5 phosphodiesterase inhibitors. J. Enzym. Inhib. Med. Chem..

[26] Cramer R.D. (2003). Topomer CoMFA: A design methodology for rapid lead optimization. J. Med. Chem..

[27] Virupaksha B., Alpana G. (2012). CoMFA QSAR models of camptothecin analogues based on the distinctive SAR features of combined ABC, CD and E ring substitutions. Comput. Biol. Med..

[28] Wang X., Pan G., Gong J., Liu X., Li H. (2016). Enhancing the Enrichment of Pharmacophore-Based Target Prediction for the Polypharmacological Profiles of Drugs. J. Chem. Inf. Model..

[29] Qiao Y., Guo S. (2005). Concise applications of molecular modeling software-MOE. Comput. Appl. Chem..

[30] Andersson C.D., Thysell E., Lindstrom A., Bylesjo M., Raubacher F., Linusson A. (2007). A Multivariate Approach to Investigate Docking Parameters' Effects on Docking Performance. J. Chem. Inf. Model..

[31] Phillips J.C., Braun R., Wang W., Gumbart J., Tajkhorshid E., Villa E. (2012). Scalable molecular dynamics with namd. J. Comput. Chem..

[32] Wang Y., Harrison C.B., Schulten K., Mccammon J.A. (2011). Implementation of Accelerated Molecular Dynamics in NAMD. Comput. Sci. Discov..

[33] Strahan G.D., Keniry M.A., Shafer R.H. (1998). NMR structure refinement and dynamics of the K+-[d(G3T4G3)]2 quadruplex via particle mesh Ewald molecular dynamics simulations. Biophys. J..

[34] Andersena H.C. (1983). Rattle: A “velocity” version of the shake algorithm for molecular dynamics calculations. J. Comput. Phys..

